# Computer-aided multiple-head 3D printing system for printing of heterogeneous organ/tissue constructs

**DOI:** 10.1038/srep21685

**Published:** 2016-02-22

**Authors:** Jin Woo Jung, Jung-Seob Lee, Dong-Woo Cho

**Affiliations:** 1 Department of Mechanical Engineering, Pohang University of Science and Technology (POSTECH), 77 Cheongam-Ro. Nam-Gu. Pohang. Gyeongbuk, 37673 Korea

## Abstract

Recently, much attention has focused on replacement or/and enhancement of biological tissues via the use of cell-laden hydrogel scaffolds with an architecture that mimics the tissue matrix, and with the desired three-dimensional (3D) external geometry. However, mimicking the heterogeneous tissues that most organs and tissues are formed of is challenging. Although multiple-head 3D printing systems have been proposed for fabricating heterogeneous cell-laden hydrogel scaffolds, to date only the simple exterior form has been realized. Here we describe a computer-aided design and manufacturing (CAD/CAM) system for this application. We aim to develop an algorithm to enable easy, intuitive design and fabrication of a heterogeneous cell-laden hydrogel scaffolds with a free-form 3D geometry. The printing paths of the scaffold are automatically generated from the 3D CAD model, and the scaffold is then printed by dispensing four materials; i.e., a frame, two kinds of cell-laden hydrogel and a support. We demonstrated printing of heterogeneous tissue models formed of hydrogel scaffolds using this approach, including the outer ear, kidney and tooth tissue. These results indicate that this approach is particularly promising for tissue engineering and 3D printing applications to regenerate heterogeneous organs and tissues with tailored geometries to treat specific defects or injuries.

Over the past three decades, tissue engineering has emerged as a particularly promising technique for reconstructing tissue defects, as well as fabricating complete organs^1^. A number of tissue engineering approaches has been developed for creating artificial organs or tissue constructs that are similar to the native tissues, and are composed of multiple cell types with three-dimensional (3D) matrix structures^2^. Many existing approaches to tissue engineering involve cell-seeded 3D biodegradable scaffolds containing biochemical and physicochemical factors to form biological tissues or organs^3^. Scaffolds are especially important for determining the exterior form of the generated tissue, and provide mechanical support and stability for pre-tissue constructs, as well as promoting formation of specific tissues^4^,^5^.

To fulfill these requirements, recent approaches to tissue engineering have focused on 3D printing technology to fabricate scaffolds with good control over the micro-architecture^6^. Such approaches to scaffold fabrication are particularly suitable for obtaining not only the required exterior form, but also an interior architecture that is capable of enhanced cell-seeding^7^, mechanical strength^8^,^9^ and oxygen and nutrients transport^10^,^11^.

Recent advances in 3D printing for tissue engineering have enabled cells to be directly printed into the desired 3D structure^6^. Rather than direct seeding of cells onto a printed scaffold, cell-encapsulated hydrogels can be printed directly to form cell–hydrogel 3D patterns. This cell-printing method has important advantages compared with conventional cell-seeding techniques, including a high cell-seeding efficiency^12^ and control over the micro-matrix formed by the printed cells^13^. Conventional inkjet-based cell-printing techniques provide 2D patterns of small hydrogel solutions droplets containing single or few cells^14^. However, creating 3D cell-patterned constructs using this approach remains a significant challenge, due to problems with slow printing speed to build 3D cell-laden constructs with general tissue size, and nozzle clogging during droplet ejection of solutions with a high cell density^6^. Dispensing-based 3D printing has also been suggested for direct 3D cell printing of cell-laden hydrogel constructs with a larger volume. Although this system generally has low resolution for making a cell pattern, the dispensing of hydrogel solution with a high flow rate allows more rapid printing of 3D cell-laden hydrogel constructs. However, stacking 2D patterns made of only a cell-laden hydrogel remains a challenge. Non-crosslinked hydrogel during the printing process can easily collapse due to its weight. Although rapid crosslinking^15^,^16^,^17^,^18^,^19^,^20^,^21^,^22^ or the use of a temporary supporting solution^23^,^24^ has been suggested for layer-by-layer assembly of cell-laden hydrogel patterns, few studies have successfully demonstrated the printing of complex and tall constructs. In addition, the only hydrogel construct with sufficient height and volume to mimic general organs and tissues can be easily deformed and broken, due to its weakness.

To overcome these limitations, we previously described a multiple-head 3D printing system for fabricating a 3D cell-laden constructs using the frames^25^,^26^,^27^,^28^. In this construct, cell-laden hydrogel solution is simply trapped between frames of thermoplastic biodegradable polymer, such as poly (ε-caprolactone) (PCL), poly (lactic-co-glycolic acid) (PLA), and poly (lactic-co-glycolic acid) (PLGA). Since the polymers would be more rigid than printed hydrogels, the frame-added construct would be more effective for supporting its original printed shape with a large volume than a construct containing only hydrogel. In addition, the degradation rate of constructs can be effectively controlled by the degradable characteristics of the frame material. Therefore, this printing approach would enable not only the printing of a large-volume 3D cell-laden construct, but also a construct offering mechanical support at defects in various organs and tissues, including hard tissues, until new tissue matrix has successfully formed.

The procedure of the suggested method involves the following steps: (i) forming a frame pattern, whereby a cross-section of the 3D model is generated by dispensing a synthetic polymer, and (ii) depositing the cell-encapsulated hydrogel solution within the frame, followed by cross-linking. Steps (i) and (ii) are iterated to obtain a 3D-printed cell-laden construct in a layer-by-layer manner. During step (ii), two or more kinds of cell/pre-gel solution may be printed into separated parts via the multiple heads to realize the desired heterogeneous tissue structure. To fabricate a complex 3D construct containing undercuts, overhangs, or empty interior spaces, a temporary support can be printed between steps (i) and (ii)^26^. Figure 1 shows an example strategy for printing 3D heterogeneous tissue constructs with the external form of the outer ear.

With manual editing of computer numerical control (CNC) code, we have fabricated cell-laden constructs with a simple cuboidal shape using the suggested multiple-head 3D printing system^26^,^27^,^28^. The results of a biological assay showed that over 90% of the cells survived the printing process and the desired tissue matrix was formed within the printed constructs. For realizing 3D construct mimicking complex structures of native tissues, this advanced 3D printing procedure should include a custom computer-aided design/computer-aided manufacturing (CAD/CAM) system that enables easy, intuitive, automatically generated CNC printing paths^29^. Here we describe an algorithm to control the four heads of a multiple-head 3D printing system to fabricate heterogeneous tissue constructs composed of four parts; i.e., a frame, two kinds of cell-laden hydrogels, and a support. An algorithm for single-head 3D printing simply involves generating the printing path of a single head; however, with the four-head 3D printing process, we must consider dividing and arranging the generated printing-path data according to the parts and sequence of the dispensed materials. Despite this complicated process, 3D CAD models of heterogeneous tissue construct may be conveniently converted into printing paths using the algorithm described here. The motion of the multiple heads of the printing system is controlled to produce the desired 3D heterogeneous structure. We show fabricated 3D-printed scaffolds for the outer ear, kidney, and tooth, formed using the printing-path generation algorithm. This demonstrates the ability to construct heterogeneous scaffolds that mimic native tissues using a multiple-head 3D printing system.

## Results

### Generation of printing paths for heterogeneous tissues

Figure 2 shows an overview of the process of generating printing paths from CAD models of the outer ear, kidney and tooth. The structure of these tissues is heterogeneous and complicated. The CAD models contained two main tissue parts and one additional part for the four-head 3D printing approach. For example, with the outer ear we have cartilage, fat and temporary undercut support parts; with the kidney we have the renal pelvis/ureter, the medulla cortex, and the temporary tubular supports; and with the tooth we have the pulp, dentin and enamel. The supports in the CAD models of the outer ear and kidney are required to realize the undercut and a tubular structure; with the tooth model, this was used as an additional tissue part of the enamel. The 3D CAD models were exported in stereolithography (STL) file format, and the algorithm was used to define 3D closed surfaces of cell-laden hydrogels 1 and 2, as well as the supports. Via the slicing process, the 3D surface data were converted into the outer contours of cross sections. The line spacing and line-width were used to generate the multi-head printing paths based on the cross-section contours. The rendered 3D images shown in Fig. 2 represent the results of the slicing algorithm and the algorithm to generate printing paths, which were free from inappropriate errors or primitives.

### Printing results

Figure 3 shows 3D printed heterogeneous tissue constructs for the outer ear, kidney and tooth. The poly (ethylene glycol) (PEG) supports in the outer ear and kidney constructs were removed, and the exterior shape and size was in excellent agreement with the 3D CAD models. The dimensions of the outer ear were 71 × 60 × 11 mm, those of the kidney were 85 × 53 × 9.5 mm, and those of the tooth were 65.5 × 38.7 × 15.5 mm. The interior architecture was also in good agreement with the 3D CAD models. The line width of the PCL frame was approximately 250 μm, and the line spacing was approximately 800 μm. The alginate hydrogel was placed between the PCL frame, and the hydrogel parts 1 and 2 appear blue and red, respectively. Video 1 shows that the motion of the print-head was controlled accurately. The printing times were 210 minutes for the outer ear, 200 minutes for the kidney, and 50 minutes for the tooth.

## Discussion

Direct printing of multiple cell lines is considered critical to mimic the microstructure and macrostructure of native tissues. Many organs and tissues contain different types of cells, micron-scale matrix structures and biological components, with specific and complicated architectures^6^. For this reason, we used a multiple-head 3D printing system to fabricate 3D free-form structures composed of different thermoplastic or soluble polymers, hydrogels and cells^25^. Using this system, we have previously demonstrated the ability to manufacture simple structures using manually generated CNC printing paths. However, fabrication of 3D free-form shapes mimicking native tissues is of limited utility without a CAD/CAM system to automatically generate CNC printing paths for multi-head printing.

To address this need, we developed an algorithm to print 3D heterogeneous tissue constructs by dispensing four different materials using a four-head 3D printing system. To provide an easy-to-use and intuitive user interface, printing paths were generated automatically as part of the design of the exterior structure of 3D models, which can be generated using commercial CAD software packages, or using bio-medical imaging systems such as computed tomography or magnetic resonance imaging. The interior architecture can be generated in an automated fashion based on a user-defined line width, line spacing, and layer thickness. The results demonstrate that the integration of this algorithm with the multiple-head tissue/organ building system (MtoBS) allowed us to fabricate 3D free-form structures, mimicking the native tissues of two heterogeneous parts. We fabricated scaffolds for an outer ear, a kidney and a tooth using the MtoBS 3D printing system. There was some infiltration and blending of the printed hydrogels at the boundaries of the parts, which is related to the fluid properties and dispensing parameters of the print-heads. As shown in Video 1, the print-heads accurately followed the generated printing-paths.

During the preparation step used to print the multiple-material constructs, the sodium alginate solutions were stained using blue and red ink to investigate the accuracy of the printing paths with different cell-laden hydrogels. In addition, cell viability after the printing of a cell-laden hydrogel between frames was evaluated using a Live/Dead assay (Invitrogen, Grand Island, NY, USA). The fluorescence microscopic image (Supplementary Figure 1) presents more than 90% of the cells remained viable in the hydrogel. These results show that we are able to control the spatial distribution of multiple components, which can be cell-laden hydrogels^30^,^31^,^32^, or hydrogels containing biological components such as cytokines, growth factors, and other biological components^33^,^34^,^35^. Furthermore, considering the synergistic effects of materials and cells, multiple-head 3D printing with the algorithm described here is expected to facilitate the development of novel tissue-engineered heterogeneous constructs with controllable 3D geometries.

The algorithm developed here currently supports heterogeneous printing of only two different hydrogels; however, many organs and tissues are composed of three or more cell types, which is essential to maintain cellular activity and physiological function. Future work will thus include increasing the number of materials that can be dispensed simultaneously to create more complex parts.

In conclusion, 3D printing technology has had a tremendous impact on tissue engineering, and has considerable potential for rapid and precise production of tailored free-form scaffolds. Over the past decade, a number of 3D printing techniques have appeared for the fabrication of internal and external structures for specific tissue generation applications. These advances require development of new algorithms enabling easy and intuitive user interface tools for the generation of printing paths from 3D CAD models.

Regeneration of heterogeneous organs and tissues, such as the liver, heart, cornea, kidney and skin, may be considered as the main goal of tissue engineering. The CAD/CAM system described here enabled the use of a multiple-head 3D printing system to construct heterogeneous tissue scaffolds with 3D free-form geometries. The resulting tissue scaffolds for the outer ear, kidney and tooth demonstrate the potential of this approach for tissue engineering to generate heterogeneous organs, or tissues with a tailored geometry to repair specific defects or damage.

## Methods

### CAD-based 3D bio-modelling

There have been several approaches to the design of 3D scaffold models, including CAD-based methods, image-based design, implicit surfaces and space-filling curves^36^. To provide a well-organized, custom 3D construct for a specific organ or tissue defect, careful design is required. CAD-based modelling is the preferred method for scaffold fabrication using 3D printing^36^, due to the simple yet powerful user-interfaces of commercial CAD software packages. The solid and surface design tools of CATIA V5 (Dassault Systemes, Vélizy-Villacoublay, France) were used here to design the exterior structure of heterogeneous tissues. The resulting models had two main tissue parts, plus a support part, as shown in Fig. 4. The resulting designs for these parts were exported individually as STL files describing the 3D surface as triangular polygons in a Cartesian coordinate system.

### Slicing algorithm

The 3D printing technique produces 3D free-form structures via vertical stacking of 2D patterns generated from cross-sections of the designed 3D model. The slicing algorithm involves calculating contours (i.e., the outer boundary) of the cross-sections of successive planes. The separation between planes is pre-determined, giving the layer thickness, and the contours are then generated from intersections between the planes and the polygons of the imported STL data. Figure 5 shows the resulting cross-section contours, together with the stacked data description.

### Algorithm to generate printing paths

**Figure 1:**
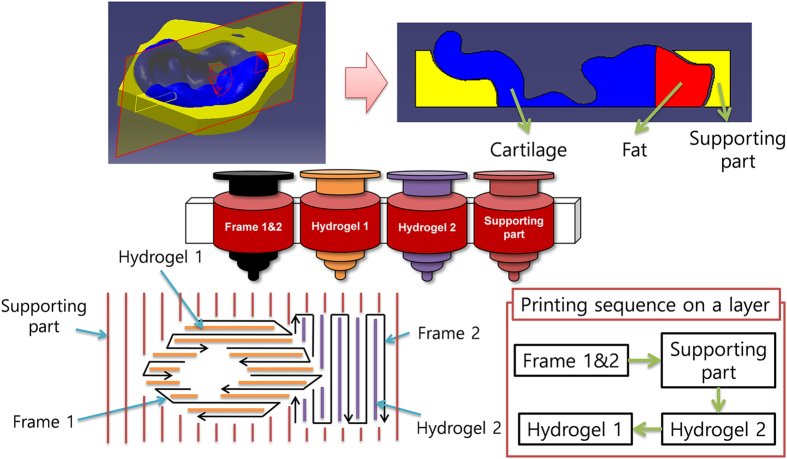
A strategy to print a heterogeneous tissue construct composed of two tissue parts and a support. In this manner, printing of outer ear, cartilage- and fat-specific hydrogels, and supporting parts is required to mimic the native tissue structure.

**Figure 2:**
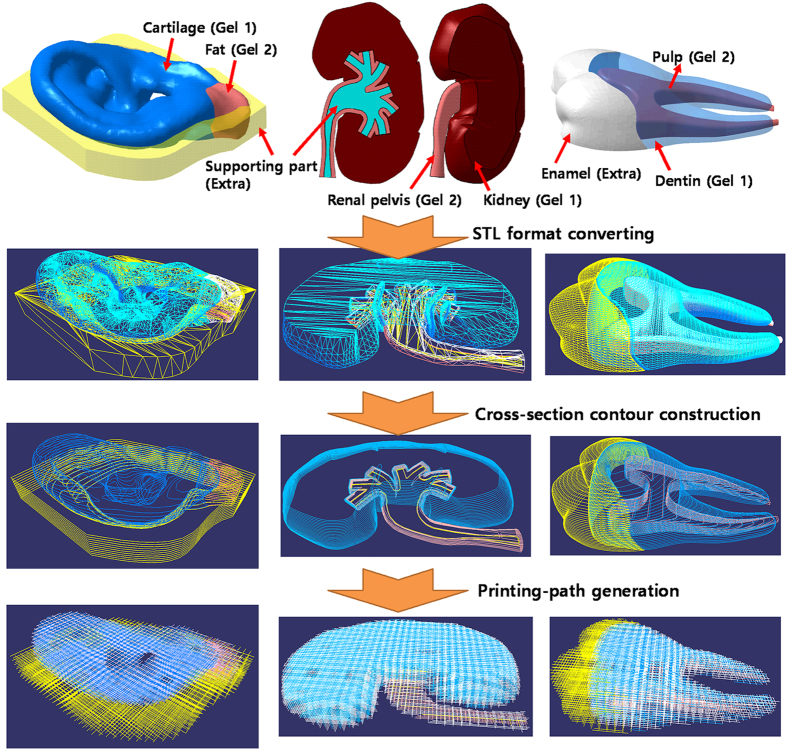
The process of generating printing paths for the outer ear (left), kidney (center), and tooth (right). The frame is shown in white, hydrogel 1 in blue, hydrogel 2 in red, and the support in yellow.

**Figure 3:**
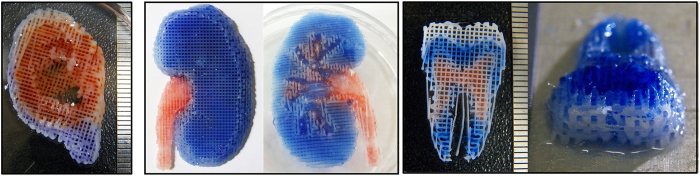
Printed structures of the outer ear (left), kidney (middle) and tooth (right), which are composed of heterogeneous tissue parts. The frame part was partially stained by the colored alginate solutions during printing.

**Figure 4:**
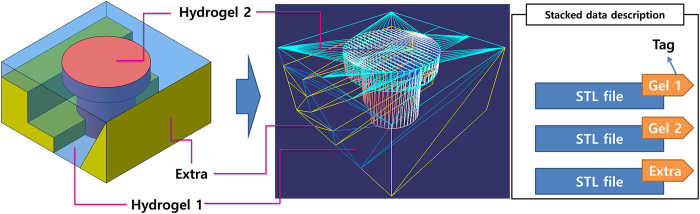
A computer-aided design (CAD) model of a heterogeneous tissue construct composed of hydrogel parts 1 (shown in blue) and 2 (shown in red), as well as the support part (shown in yellow), together with the stacked stereolithography (STL) format description of the model.

**Figure 5:**
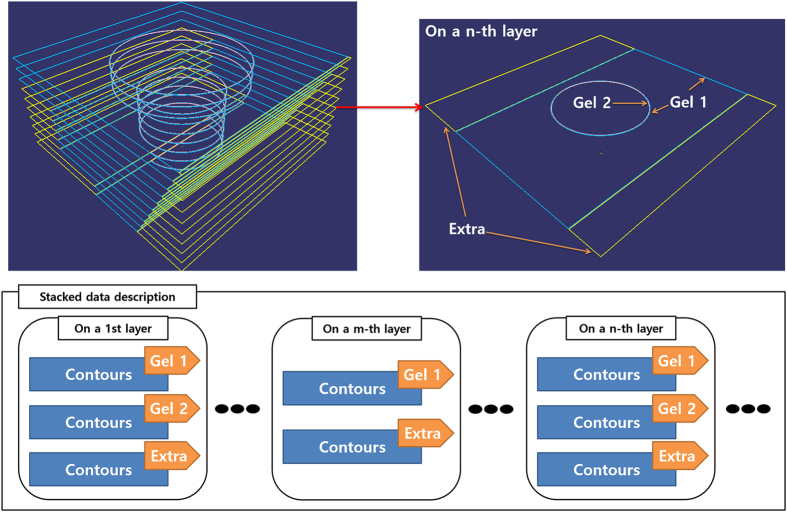
Cross-section contours generated from the imported STL data (left), with a single-layer contour (right). Hydrogel part 1 is shown in blue and hydrogel part 2 in red. The support is shown in yellow.

**Figure 6:**
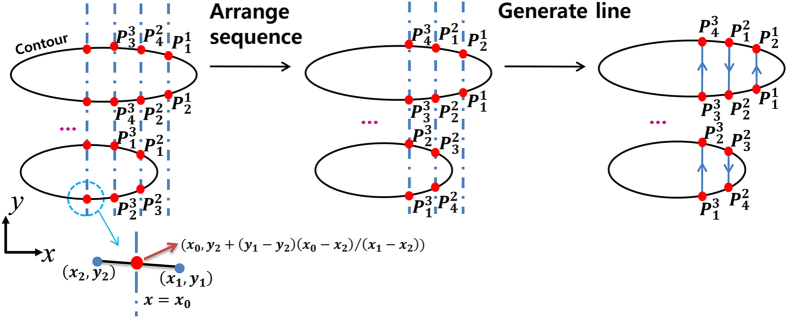
The generation and arrangement of printing paths. The printing paths are composed of lines between points 1, 2, …, *n*, and printhead moves starting from point 1. The printing paths are arranged in a zigzag manner to minimize the total length of the printing path.

**Figure 7:**
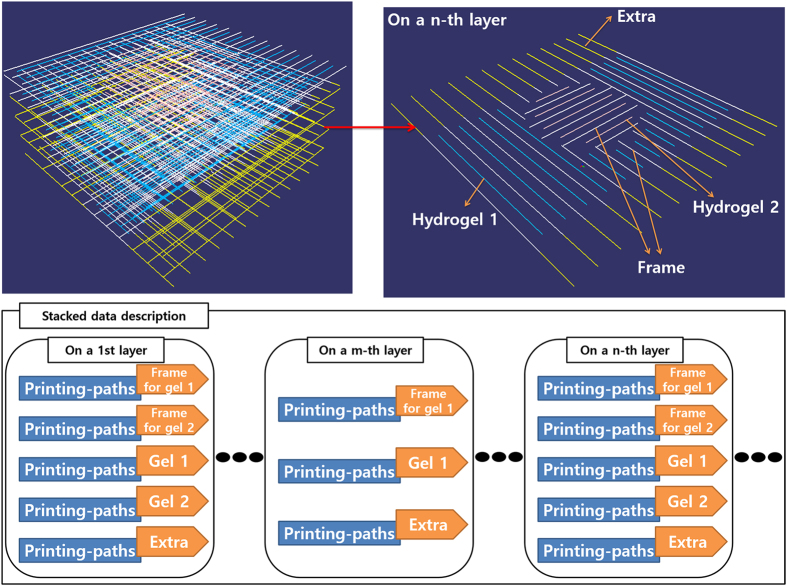
The printing paths of the frame (white), the two hydrogels (blue and red), and the support (yellow), together with descriptions of the printing paths for each layer. The frame for printing hydrogel 1 is perpendicular to that for hydrogel 2 to minimize mixing.

The printing path for the frame is defined based on the cross-section contours of the two cell-laden hydrogel parts, as follows. First, successive parallel lines determined by the line spacing are placed on the contours. With each layer, the lines for cell-laden hydrogel part 1 are perpendicular to those of cell-laden hydrogel part 2, which minimizes direct contact and mixing between the cell-laden hydrogels (see Fig. 1). The end points of the printing paths are then determined by calculating the intersection between the lines and the contours. To minimize the total length of the print-head trajectory, the printing path is determined as shown in Fig. 6. Here the lines on each layer are allocated into a single group of printing paths to define the frame. Similarly, the printing path for the support is calculated from the contours belonging to that part. Finally, the printing paths of the two cell-laden hydrogel parts are placed between the lines that make up the printing path of the frame. Figure 7 shows the resulting printing paths.

### CNC code-generation algorithm

**Figure 8:**
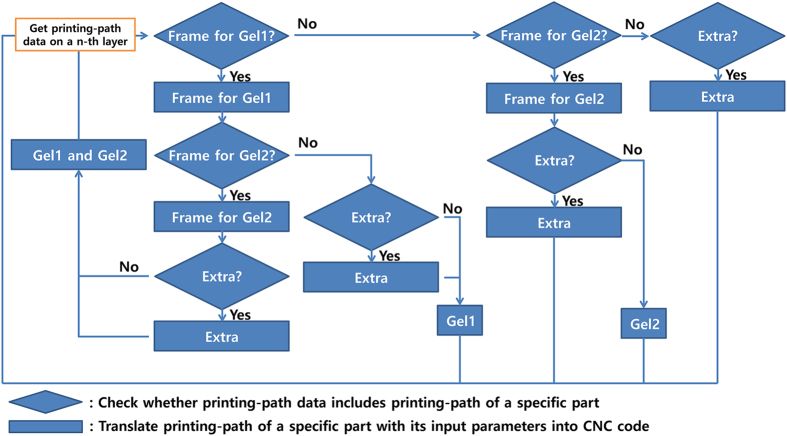
Process for translating the printing paths into CNC code, with specific head-control parameters. The motion of each head can be controlled separately using different parameters for the material that is to be dispensed.

The final step of the algorithm is the generation of commands to control the four-head printing system, based on user-defined parameters to control the head (i.e., the speed of the head and the dispensing pressure). This algorithm is composed of the following two steps (see Fig. 8): (i) check which part the printing path corresponds to (i.e., frame, cell-laden hydrogels 1 and 2, or the support), and (ii) translate the two end points of the line based on the user-defined parameters for dispensing a specific material into CNC code to control the 3D printing system.

### Overview of MtoBS

The MtoBS is a custom six-head 3D printing system^25^, and has been used to fabricate various hybrid constructs for tissue engineering applications. The MtoBS is composed of a motion control system, which enables the nozzle to accurately translate throughout the 3D workspace, and a dispensing system, which includes six dispensers. The *XY* motion system is composed of a linear motor, a linear encoder and a linear guide to control accurately the position of the head in the *x-y* plane, with an accuracy of ±2.4 μm and repeatability of ±1.0 μm. The *Z*-axis motion of the dispensing heads is controlled individually using an AC servomotor with a rotary encoder, giving an accuracy of ±5.0 μm and repeatability of ±5.0 μm. The six heads of the dispensing system are connected to the *Z*-axis of the motion control system. Two printheads contain a heating block to melt the thermoplastic biomaterials, which are sprayed out of a narrow nozzle^25^. The temperature and pressure of the two heads can be controlled to 150 °C and 650 kPa. The remaining four heads have a Peltier block for temperature control in the range −5 °C to +100 °C to maintain cell viability, printability and to achieve thermal cross-linking of the hydrogel solution. The cooled hydrogel solution can be dispensed with a resolution of 1 μL using a plunger system. The MtoBS therefore allows printing of 3D hybrid pre-tissue constructs composed of various biomaterials with micron-scale precision of all six heads.

### Material and printing preparations

Four heads (two pneumatic pressure-type heads and two plunger-type heads) were used to fabricate the bio-mimetic constructs (i.e., outer ear, kidney and tooth). First, PCL (Sigma-Aldrich, St Louis, MO, USA, 45 000 < *M*_*w*_ < 60 000), PEG (Sigma-Aldrich, *M*_*w*_ = 20 000), and a solution of 4% medium-viscosity sodium alginate (Sigma-Aldrich) were prepared. There were two different color alginate solutions, which were stained using a small volume (0.1% of the total alginate solution volume) of blue and red inks (Rotring GmbH, Hamburg, Germany). Granules of PCL (for the tooth) or PCL and PEG (for the outer ear and kidney) were inserted separately into two syringes installed for the pneumatic pressure-type heads, and two syringes containing the two colored alginate solutions were prepared and installed for the plunger-type heads. Prior to printing the 3D constructs, the separations between the four heads were measured using a digital microscope (AM3113T Dino-Lite Premier, AnMo Electronics Corporation, Hsinchu, Taiwan), and used as input parameters to calculate the printing paths.

### Printing of 3D heterogeneous tissue constructs

Printing paths for the kidney, outer ear and tooth heterogeneous tissue construct were generated with a PCL line width of 250 μm, a PCL line distance of 800 μm, a PEG line width of 250 μm, a PEG line distance of 250 μm, and a layer thickness of 600 μm. The PCL and PEG granules were inserted into the syringes, melted by heating to 85 °C, and extruded from a 250-μm-diameter nozzle at a pressure of 500 kPa. The alginate solution was dispensed from a 22-gage needle with a flow rate of approximately 0.88 μl sec^−1^. The alginate solution was cross-linked using 100-mM CaCl_2_ solution for 10 minutes, and the PEG part was dissolved using distilled water at room temperature. After this final step, the remaining parts were carefully removed from the print-bed.

## Additional Information

**How to cite this article**: Jung, J. W. *et al.* Computer-aided multiple-head 3D printing system for printing of heterogeneous organ/tissue constructs. *Sci. Rep.* **6**, 21685; doi: 10.1038/srep21685 (2016).

## Supplementary Material

Supplementary Information

Supplementary Video 1
