# Supplementary material for: Computer-aided multiple-head 3D printing system for printing of heterogeneous organ/tissue constructs
Source: Sci Rep. 2016 Feb 22;6:21685. doi: 10.1038/srep21685 (PMC4761951; doi:10.1038/srep21685)
Supplement: Supplementary Information [file srep21685-s1.doc]

Supplementary Information

**Computer-aided multiple-head 3D printing system for printing of heterogeneous organ/tissue constructs**

Jin Woo Jung^1,+^, Jung-Seob Lee^1,+^, and Dong-Woo Cho^1,*^

^1^ Department of Mechanical Engineering, Pohang University of Science and Technology (POSTECH), 77 Cheongam-Ro. Nam-Gu. Pohang. Gyeongbuk. Korea 37673

^*^ corresponding author. [dwcho@postech.ac.kr](mailto:dwcho@postech.ac.kr); Tel: +82-10-9357-2171

^+^ these authors contributed equally to this work

**Supplementary Figure 1**

**Methods**

The embryonic fibroblast cell line NIH/3T3 was used for the evaluation of cell viability within the cell-laden construct. A solution of 4% sodium alginate with (5×10^6^ cells/ml) NIH-3T3 and 1x DMEM–high–glucose medium was inserted into a syringe installed in the plunger-type printhead. Granules of PCL were introduced into the pressure-type printhead. The PCL was heated at 85°C and extruded for frame printing. Cell-laden alginate hydrogel solution was placed between the frames. The complete construct with a cuboidal exterior shape was immersed in 100 mM CaCl_2_ for 10 min for crosslinking of the alginate solution.

Cell viability and positioning were observed using the Live/Dead assay kit (Invitrogen, Grand Island, NY, USA), in accordance with the manufacturer's protocol. After the printing process, the construct was placed in PBS solution containing calcein AM and ethidium homodimer (EthD-1) and incubated for 20 min at 37°C. Live and dead images were acquired using a confocal microscope (Olympus Confocal FluoView 1000, Tokyo, Japan). Living cells were identified by green fluorescence and EthD–1 stained dead cells by red fluorescence.

**Results and discussion**

The dimensions of the construct were 8.4 × 8.4 × 3 mm. A fluorescence image showed that most of the cells had survived printing of the complete construct. Although PCL in the syringe is extruded out of the nozzle at 85°C, the PCL frame pattern would rapidly cool and have low thermal capacity because the line of the pattern is very thin. Therefore, heat diffusion from the extruded PCL to the hydrogel would not lead to cell death or damage.


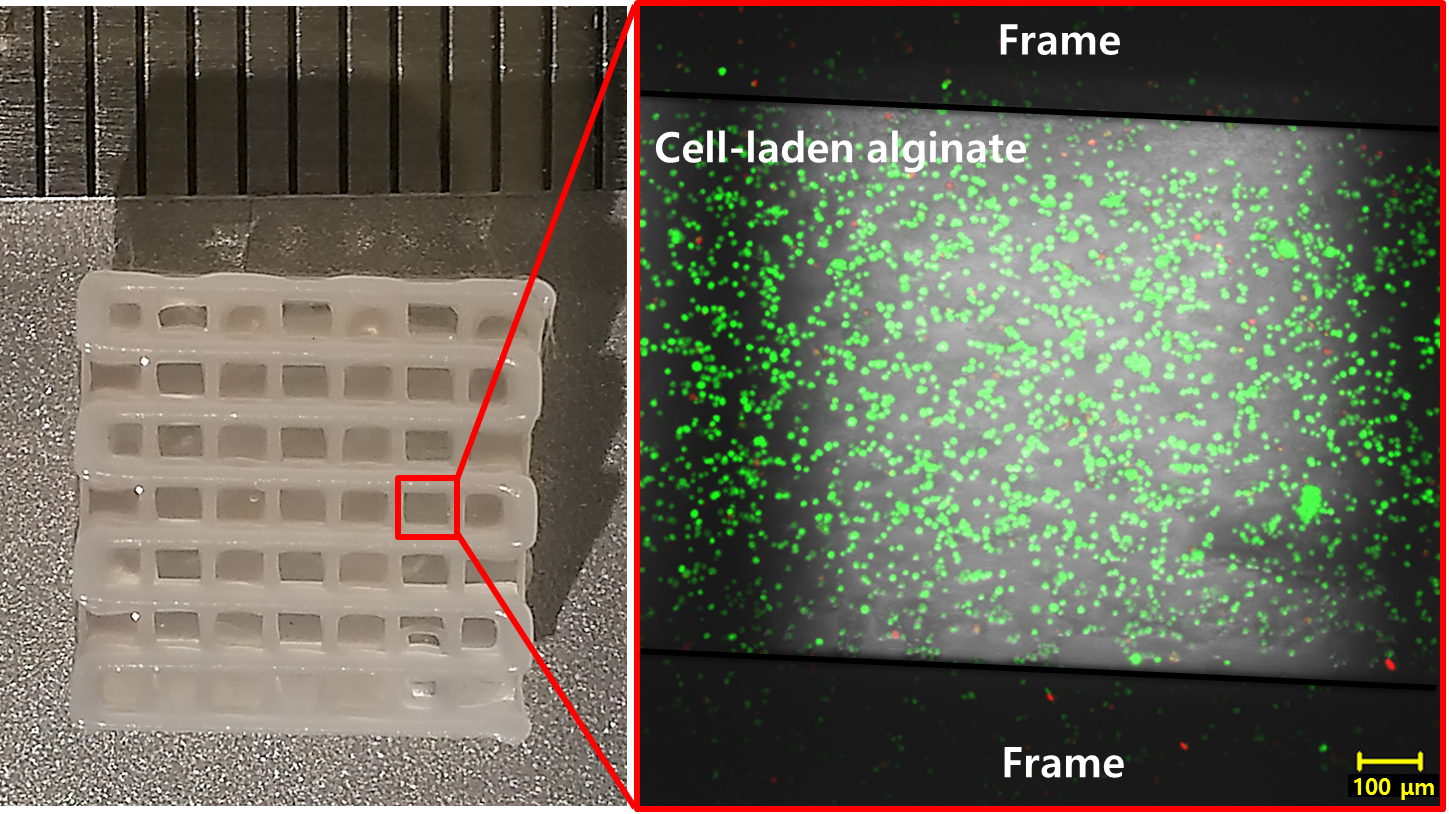


**Supplementary Figure 1.** Cell-laden hydrogel construct (left) and fluorescence microscopy image (right). Green and red colors indicate living and dead cells, respectively. Cells within the hydrogel were placed in the PCL framework and most of the cells survived the printing process.
